# Sulfadiazine Plus Pyrimethamine Therapy Reversed Multiple Behavioral and Neurocognitive Changes in Long-Term Chronic Toxoplasmosis by Reducing Brain Cyst Load and Inflammation-Related Alterations

**DOI:** 10.3389/fimmu.2022.822567

**Published:** 2022-04-27

**Authors:** Barrios Leda Castaño, Andrea Alice Silva, Lina L Hernandez-Velasco, Ana Paula Da Silva Pinheiro, Daniel Gibaldi, José Roberto Mineo, Neide Maria Silva, Joseli Lannes-Vieira

**Affiliations:** ^1^Laboratory of Biology of the Interactions, Oswaldo Cruz Institute/Fiocruz, Rio de Janeiro, Brazil; ^2^Multiuser Laboratory for Research Support in Nephrology and Medical Sciences, Federal University Fluminense, Niterói, Brazil; ^3^Faculty of Basic Sciences, University of Santiago de Cali, Cali, Colombia; ^4^Institute of Biomedical Sciences, Federal University of Uberlândia, Uberlândia, Brazil

**Keywords:** *Toxoplasma gondii*, anxiety, depression, hyperactivity, memory loss, neuroinflammation, cytokines

## Abstract

*Toxoplasma gondii* infects one-third of the world population. For decades, it has been considered a silent lifelong infection. However, chronically *T. gondii*-infected persons may present psychiatric and neurocognitive changes as anxiety, depression, and memory loss. In a model of long-term chronic infection, behavioral alterations parallel neuroinflammation and systemic high cytokine levels, and may reflect brain cyst load. Recent findings support that in chronic infection an active parasite-host interplay involves an immune-mediated control of tissue cysts. Here, we tested the idea that etiological treatment in chronic phase may add advantage to intrinsic immune-mediated cyst control and impact behavioral changes. Thus, we combined sulfadiazine-plus-pyrimethamine (S+P), the first-choice therapy for toxoplasmosis, to study the association of brain cyst load and biological processes related to the immune response (neuroinflammation, blood-brain barrier -BBB- disruption and serum cytokine levels), with behavioral and neurocognitive changes of long-term chronic infection. Female C57BL/6 mice (H-2^b^) were infected (5 cysts, ME-49 strain) and treated with S+P from 30 to 60 days postinfection (dpi), compared with vehicle (Veh)-treated and noninfected controls. At endpoints (pre-therapy, 30 dpi; S+P therapy, 60 dpi; after ceased therapy, 90 dpi), independent groups were subjected to behavioral tests, and brain tissues and sera were collected. Multiple behavioral and neurocognitive changes were detected in the early (30 dpi) and long-term (60 and 90 dpi) chronic infection. S+P therapy resolved locomotor alterations, anxiety, and depressive-like behavior, partially or transiently ameliorated hyperactivity and habituation memory loss. Analysis after therapy cessation showed that S+P therapy reduced the number of stimuli required for aversive memory consolidation. S+P therapy resulted in reduced brain cyst load, neuroinflammation and BBB disruption, and lowered systemic Th1-cytokine levels. Correlation analysis revealed association between IFNγ, TNF and MCP-1/CCL2 serum levels, brain cyst load and behavioral and neurocognitive alterations. Moreover, principal-component analysis (PCA-2D and 3D projections) highlighted distinction between clusters (noninfected; Veh-treated and S+P-treated infected). Thus, our data suggest that S+P therapy added gain to intrinsic brain cyst control and, direct or indirectly, ameliorated inflammation-related alterations, traits associated with behavioral and neurocognitive alterations.

## Introduction

One-third of the world population is seropositive for *Toxoplasma gondii* (1). This extremely successful protozoan parasite is the etiological agent of toxoplasmosis, a disease of two phases: (i) the acute phase characterized by the presence of circulating tachyzoite forms and (ii) the chronic phase, when cysts containing bradyzoite forms are detected in tissues (2). *T. gondii* may invade any cell type as well as any body tissues, showing, however, tropism for the central nervous system (CNS) (1, 3). Although the success of therapy is guaranteed during the acute phase, there is no etiological treatment capable of eliminating the parasite in the chronic infection, as cysts are mostly resistant to currently available drugs (4). The folate pathway, involved in DNA synthesis with participation of the enzymes dihydrofolate reductase and dihydrofolate synthetase, is the main target of the available anti-*Toxoplasma* drugs (4). Combined sulfadiazine (S) plus pyrimethamine (P) therapy (S+P) is the first-choice treatment for toxoplasmosis (4). P acts on the parasite dihydrofolate reductase but is unable to distinguish it from the human enzyme. More, P acts synergistically with S, blocking dihydrofolate synthetase, an enzyme indispensable for folate biosynthesis (4). The pathways underlying the S therapeutic effect are still unknown, but changes related to the host metabolism are likely to occur (5). Corticosteroids used in encephalic and ocular toxoplasmosis to minimize tissue damage caused by inflammation (6), may, however, suppress the specific immune response against the parasite and increase the severity of the disease, thus highlighting the role of intrinsic immune response to parasite control (6). Indeed, fulminant ocular toxoplasmosis may occur after application of corticosteroids in monotherapy or combined with antiparasitic treatment (7).

In the acute infection, the robust Th1 immune response, characterized by production of proinflammatory cytokines, as interferon-gamma (IFNγ) and tumor necrosis factor (TNF) is required for an efficient antiparasitic response (8). Although the proinflammatory response is sustained during the chronic phase of infection (9, 10), the parasite evades the immune response, remaining in tissues as cysts, mainly in the CNS throughout the host life, in an apparent silent state considered harmless to the host (8). Studies carried out in acutely and chronically *T. gondii*-infected perforin-deficient mice revealed a perforin-dependent cytolytic function that contributes to the control of brain cysts during the chronic phase of the infection (11). Furthermore, CD8^+^ T-cells favor effective parasite control in the CNS, thus limiting the development of encephalic toxoplasmosis (12). Additionally, an active protective innate and adaptive immune response, mediated by phagocytes and perforin-dependent CD8^+^ T cells, against mature cysts, was reported (10). Thus, questioning the concept of the existence of “quiescent infection” in the chronic phase of *T. gondii* infection, which has been accepted for many years. On the other hand, psychiatric and behavioral changes such as schizophrenia (13, 14), obsessive-compulsive disorders, personality and bipolar disorders (14), depression (15) and alteration of neurocognitive functioning (16) have been associated with chronic *T. gondii* infection in human beings. Several studies investigated the influence of *T. gondii* infection in behavioral and neurocognitive disorders using animal models, replicating aspects present in humans as locomotor alteration (17), anxiety-like disorder (18–22), hyperactivity (20, 22–25), and also alterations in aversive memory consolidation (26, 27), spatial memory loss (28), and long-term memory impairment (21).

The mechanisms underpinning behavioral and neurocognitive changes are not unveiled. In humans, mental disorders with behavioral and neurocognitive alterations have been linked to neuroinflammation and systemic inflammation in infectious and noninfectious diseases (29, 30). Although mostly linked to neuroinflammation, as in Alzheimer disease (29), behavioral and neurocognitive alterations can also be detected in the absence of neuroinflammation (31, 32). Importantly, behavioral changes were described after administration of IFNα to hepatitis virus-infected patients (33). More, systemic inflammatory profile has been associated with behavioral abnormalities in major psychiatric illnesses (30). In mice chronically infected with *Trypanosoma cruzi*, depressive-like behavior was associated with increase in systemic TNF levels and, moreover, reversed by anti-TNF therapy, but independent of neuroinflammation (31). Thus, a more complex network of biological interactions may underpin the onset and progression of behavioral and neurocognitive changes in noninfectious and infectious conditions. In a mouse model of *T. gondii* infection, neuroinflammation was raised as determinant factor for hyperactivity independent of brain cyst load (23). In ME-49 *T. gondii*-infected male B6CBAF1/J mice, behavioral alterations were associated with inflammation-related processes and a continuous increase in cyst load in the CNS (34). Conversely, a recent study showed that in female C57BL/6 mice ME-49 infection evolves from the early to the long-term chronic phase with reduction of the number, suggestive of gradual infection control, as immune response is established (22). However, multiple behavioral changes (anxiety-like disorder, depressive-like behavior and hyperactivity) were detected in the early and long-term chronic infection (22). Further, these behavioral alterations occur in a scenario of upregulation of inflammatory cytokines and chemokines in the CNS, neuroinflammation, blood-brain barrier (BBB) disruption, and increase in systemic Th1 cytokine levels (22). Altogether, these findings support the participation of inflammation-related processes in behavioral changes, probably reflecting brain cyst load, still undisclosed. In the present work, we tested the idea that the use of the combined etiologic S+P therapy when *T. gondii* infection progresses from the initial to its long-term chronic phase may be advantageous to the intrinsic immune-mediated cyst control and impact behavior and neurocognitive changes. To test this idea, female C57BL/6 mice infected with the cystogenic ME-49 type II strain received S+P therapy from early to late chronic phase, from 30 to 60 days postinfection (dpi), and were compared with matched vehicle (Veh)-treated and noninfected (NI) controls. Based on a previous kinetic study showing the establishment of the early (30 dpi) and long-term (60 and 90 dpi) chronic infection (22), mice were analyzed at endpoints pre-therapy (30 dpi), S+P therapy (60 dpi) and after ceased therapy (90 dpi). We used standardized tests to evaluate locomotor/exploratory activities, anxiety-like disorder, depressive-like behavior, and spatial and aversive memory. Further, we addressed possible biological factors involved, thus evaluating brain cyst load, neuroinflammation, BBB disruption and systemic cytokine levels. Finally, correlation and principal-component analysis (PCA-2D and 3D projections) were performed.

## Material and Methods

### Ethics Statement

The experimental procedures were performed in accordance with the recommendations of the Guide for the Care and Use of Laboratory Animals of the National Council for Animal Experimentation. The Animal Use Ethics Committee of Oswaldo Cruz Institute/Fiocruz approved all procedures performed in this study (license L014/2018). All the data presented were obtained from two independent experiments registered in the Experiment Record Book #73, LBI/IOC-Fiocruz.

### Experimental Design

A total of 153 female mice with 3 to 4 weeks of age of the lineage C57BL/6 (H-2^b^), was provided by the Institute of Science and Technology in Biomodels (ICTB) of the Oswaldo Cruz Foundation and housed in the Experimental Animal Facility (CEA-CF/IOC unit), under specific pathogen-free conditions. Mice were randomly grouped into groups of 3 to 5 animals and placed in a polypropylene cage lined with pine sawdust and enriched with an igloo, kept in microisolators, and received water and grain-based *ad libitum*. Upon arrival at the Experimental Animal Facility, the animals remained unhandled in the cages for 15 days to facilitate adaptation to the new environment. The environmental conditions were controlled with temperature of 22 ± 2°C and a 12-hours cycle of light and dark. After the adjustment period, mice were infected and analyzed according to the experimental protocols (**Supplementary Figure S1**). Upon arrival, the experimental groups were randomly formed. The cages were numbered and classified for experimental infection, and mice were analyzed at the indicated endpoints. The following groups were defined as Experiment 1: (i) Pre-therapy, 30 dpi: 5 NI controls and 12 infected; (ii) Therapy, 60 dpi - mice treated between 30 and 60 dpi, subdivided into two groups (a) Veh-treated: 6 NI controls and 15 infected and (b) S+P-treated: 6 NI controls and 12 infected; and (iii) Ceased Therapy, which consisted of mice evaluated 30 days after therapy cessation (90 dpi), subdivided into 2 groups (a) Veh-treated ceased: 6 NI controls and 24 infected and (b) S+P-treated ceased: 6 NI controls and 21 infected. Experiment 2 (i) Pre-therapy (30 dpi): 4 NI controls and 10 infected; and (ii) Therapy (60 dpi), subdivided into 2 groups (a) Veh-treated: 4 NI controls and 10 infected (b) S+P-treated: 4 NI controls and 8 infected.

### *Toxoplasma gondii* Infection and Clinical Follow-Up

Mice were infected by gavage with 0.2 mL of pyrogen-free saline (BioManguinhos, Fiocruz) containing five cysts of the cystogenic ME-49 *T. gondii* strain (35), provided by Dra. Neide Maria da Silva (ICBIM, UFU) and maintained at the Laboratory of Biology of the Interactions (LBI-IOC) by serial passages in female C57BL/6 mice every 60 days. Mice were monitored daily. Weight was evaluated weekly using a mouse precision scale (Sartorius ED623S Milligram Scale, OCE), and following clinical signs were registered: piloerection, apathy, prostration, mobility, posture, aggressive behavior, pain, weight loss and mortality. Signs of pain, isolation from the group, loss of body weight greater than 30% of initial weight, fight injuries, ataxia and immobility were the criteria established to guide the decision-making endpoint for ethical recommendations. In view of the pandemic scenario, experiments to confirm our initial kinetic studies were combined and simultaneously performed with our therapy studies. Thus, results obtained in pre-therapy and vehicle-treated infected mice groups were used to compose the kinetic studies shown in our previous article (Castaño et al., 2021), considering the following parameters: muscle strength, time in the central zone, immobility in the TST, immobility in the FST, number of cysts in the CNS, relative brain weight, and concentration of EB in the brain.

### Therapy

Groups of 4 to 6 sex- and age-matched NI controls were submitted to Veh administration by gavage with 0.1 mL of apyrogenic vaccine-graded water (BioManguinhos, Fiocruz). Groups of 8 to 24 *T. gondii*-infected mice were treated for 30 consecutive days using 0.1 mL of Veh or 0.1 mL of Veh containing sulfadiazine (S, 100 mg/Kg, Sulfazina^®^, Sobral) and pyrimethamine (P, 4 mg/Kg, Daraprim, Farmoquímica S/A), as described previously (36, 37).

### General Conditions for Behavioral Tests

In order to increase familiarity and minimize stress, the environment where the behavioral tests were performed remained under controlled light conditions (cycles of 12-hours of light and 12-hours of dark) at a temperature of 22 ± 2°C and a noise level of approximately 40 dB produced by an air conditioner. All behavioral tests were performed between 8:00 am and 4:00 pm and recorded using a DSC-DVD810 video camera (Sony). Independent experimental groups (pre-therapy, therapy and ceased therapy) were subjected to behavioral tests at the endpoints. No mouse was subjected to the same test more than once, but a mouse was subjected to different tests to reduce the number of mice used in the present study. Behavioral tests were performed from the least stressful to the most stressful: (i) open field test (OFT), (ii) habituation memory test (iii) grip strength meter test (GSMT), (iv) tail suspension test (TST), (v) forced-swimming test (FST) and (vi) aversive shock evoked test (31). After testing each mouse, the device was cleaned using 70% alcohol and dried with gauze between sessions to eliminate odors and traces of the mouse tested previously.

### Open Field Test and Habituation Memory Test

The open field apparatus consists of a 60 cm acrylic cubic box, with white walls and the floor divided by black lines into 49 equal squares, where the animal is exposed to an environment without aversive or rewarding stimuli. The OFT is a straight-forward test to investigate activity or exploratory behavior and assess locomotor impairment in animal models of neuromuscular disease (38), anxiety-related behavior (39), and memory of habituation (40) in rodents. The mouse was allowed to explore freely the open field for 5 minutes for two consecutive days (day one = training; day two = test session). The training session in the open field is used to assess exploratory activity and/or locomotion and anxiety. The exploratory activity was evaluated as the number (units) of vertical activity (rearing behavior). Immobility time (s) in total time was registered. The distance traveled (cm) was calculated by the number of lines crossed during the total time. Speed or velocity was estimated as time spent crossing the lines (cm/s). To assess anxiety, we assayed the time taken exploring the central area (s). Once mice are exposed to a new environment, they prefer to be close to walls or peripheral areas, rather than being exposed to the central, more exposed area of the field, which means danger. As time elapses, anxiety levels decrease due to habituation and the mouse ventures to explore the central area. A shorter distance traveled in the central area and or less time exploring that area is an indication of an anxious behavior (39, 41). The test session is used to evaluate the habituation memory after the training session (24 hours later). The long-term memory test was performed, in which the procedure was repeated. The memory retention was evaluated by counting the number of total lines crossed on the test session (40) and the individual baseline differences were corrected using the discrimination index (DI) to compare behavior during training and test sessions, as follows: number of crossed lines crossed at day 2/(number of crossed lines at day 1 + number of lines at day 2), as described (42). DI > 0.6 indicates impairment of memory habituation. The analyzed data were discarded or considered outlier when the animal did not interact with the test in any of the sessions. The 5 min of the training and test session were recorded using a digital video camera (Sony).

### Grip Strength Meter Test

The grip strength test is a non-invasive method used to measure neuromuscular function as maximum muscle strength of the mouse limbs (43). We used the grip strength meter apparatus (EFF 305, Insight) to the GMST that consist in a grid connected to a sensor that measures the peak of traction (in gram-force). Three attempts were made in a succession of 15s, when the mouse was lightly pulled by the tail for 2-3 seconds. All grip strength values obtained are normalized by the mouse body weight.

### Tail Suspension Test

The tail suspension apparatus (Insight) consists of a box measuring 61.10 cm wide by 55.40 cm high, divided into four equal compartments (15.28 cm) and an aluminum suspension bar placed horizontally at the top, where the animal is hung by the tail with the help of a paper tape. This internal compartment division allows the animals not to touch the other walls of the compartment or observe each other. Hollow cylinders of transparent polycarbonate (4 cm long, 1.6 cm outside diameter, 1.3 cm inside diameter, 1.5 grams) are placed in the mouse’s tail to prevent climbing behavior (44). The test is based on the principle that animals placed in a moderately stressful or uncomfortable situation and without escape, will develop an immobile posture or apathetic behavior, indicative of depressive-like behavior (45). The test lasted for 5 min, once the mouse is suspended from the tail it reacts with active movements such as shaking, swinging vigorously, torsion or jerking the body, and reaching out in an attempt to escape the circumstances. When they begin to tire out, their movements become subtler. The immobility time was recorded. The movements of their front legs alone, body sway from previous movements, passive oscillations and total absence of movement are considered immobility.

### Forced Swimming Test

This test was used to assess behavioral impulsivity (46). The forced swimming test consists of a cylinder of 35 cm high and of 25 cm in diameter, which is filled with 20 cm of clean water (25 ± 1°C). Mice were individually placed inside the cylinder carefully on the surface. The animal was checked for its ability to float or not, then floating mice were maintained for 6 min in the test and non-floating mice were excluded from the test. The first 2 minutes of the test were considered habituation and the total duration of immobility was recorded during the last 4 minutes (47). Immobility was defined as the time during which the mouse remained passively floating, made no attempt to escape and showed only slow movements to keep its head above water. Strong movements with the forelimbs against the cylinder walls and swimming were considered active movements. The water was changed before introducing each animal. After the test, the animal was dried with gauze and returned to its cage.

### Aversive Shock Evoked Test

The inhibitory avoidance apparatus (EP 104MR, Insight) consisted of an aluminum box (35 x 28 x 50 cm) epoxy-painted, with acrylic front door (2 mm), whose floor consisted of parallel stainless-steel bars (3 mm diameter) spaced 1 cm apart. A 7 cm wide x 2.5 cm high platform was placed on the floor of the box against the wall on the right-hand side of the compartment. The procedure was modified from the test above (48) and consisted of 3 sessions: (i) pre-exposure session, mice were placed on the platform and allowed to explore the box freely for 1 min with no aversive stimuli; (ii) training session, which was held 2 hours after pre-exposure. Mice were placed on the platform and the latency to descend to the grid with all four paws was measured with a stopwatch. Immediately after stepping on the grid with all four paws, the animals received an aversive stimulus, produced by a 3-second electrical stimulus (0.6 mA) to the paws. The process was repeated until the aversive memory was acquired, which was considered as a descent latency greater than 120 sec. The number of stimuli needed for memory acquisition was counted to assess memory consolidation; (iii) 24 hours after the training session, the test session took place. The animal was placed again on the platform and the latency to descend was timed, no aversive stimuli were administered, and the reduction latency (maximum of 120 seconds) was used to measure memory retention. Increase in latency during testing was considered improved memory index and vice-versa.

### Determination of Blood-Brain Barrier Integrity

Permeability of the BBB was evaluated using the Evans blue (EB) dye, as described previously (49). For this, mice were intraperitoneally sedated with Diazepam (20 mg/Kg). After 15 or 20 min of sedation, 200 µL of Evans Blue dye (Sigma-Aldrich) diluted in a 1% pyrogen-free saline solution (BioManguinhos, Fiocruz) were administered *via* the orbital plexus, anesthetized previously with the application of topical eye drops. After 2 hours, mice were physically restrained and the maximum blood volume was collected by the orbital plexus, after local anesthesia with topical eye drops. Mice were euthanized at endpoints (pre-therapy, therapy and ceased therapy) using CO_2_ inhalation, followed by decapitation. According to a previously described protocol (50), the brains were collected, weighed and sagittally sectioned. Hemi-brain was placed in 1.5 mL Eppendorf tubes containing 500 µL of a 10% formalin for 10 days to extract the EB and the eluate from each hemi-brain was collected and analyzed by spectrophotometry at 620 nm (50). The dye concentration in each sample was determined by means of a standard curve, with serial dilution in the following concentrations: 3 µg/mL; 1 µg/mL; 0.3 µg/mL; 0.1 µcg/mL; 0.03 µg/mL and 0 µg/mL (diluent). The final concentrations were calculated for the whole brain.

### Evaluation of Cysts Number and Diameter

Mice were euthanized at the corresponding analysis points. Each brain collected was weighed, sagittally sectionized, and hemi-brain included in a 1.5 mL of phosphate-buffered saline (PBS), and then initially macerated using a 5 mL syringe connected to an 18G hypodermic needle, making gentle movements of aspiration and discarded after tissue disintegration. In order to homogenize the smaller particles, the process was repeated using a 21G hypodermic needle, until a homogenate was obtained. A 20 µL aliquot of the homogenate was evaluated in duplicate by light microscopy to determine the number of cysts present in the brain. The diameter of the cysts was measured using the digital morphometric device NIS Elements BR version 4.3 of the software (Nikon Co., Japan). The measurements obtained were grouped by classes to determine the frequency distribution of the length of the diameter of the cysts. The number of occurrences in each class was counted to understand the behavior related to the size of the cysts throughout the infection.

### Histopathology

Encephala were collected, weighed and sagittally cut, and then a cerebral hemisphere was fixed in a 10% buffered formalin in saline solution for 10 days, dehydrated and embedded in paraffin. Two sections of 4 to 6 μm thick sagittal sections were prepared and stained with hematoxylin and eosin. The slides were scanned using the Motic infinity 100 Scanner and viewed using the VM-Motic Digital Slide Assistance software, version 1.0.7.46. Histopathological changes were analyzed in blind by two independent observers. The number of inflammatory mononuclear cells infiltrating in the CNS areas (cortex and hippocampus) were counted in two sections per mouse, three randomly taken mice per analyzed group. Data were normalized and are shown as the number of inflammatory cells per 200 nm^2^.

### Determination of Cytokines in Sera by CBA

After injection of EB dye to analyze BBB integrity (described above), blood was collected through the orbital plexus, after local application of anesthesia eye drops. The collected blood was centrifuged to obtain serum, divided into aliquots, and stored in a freezer at -80°C until use. To measure serum cytokine levels, the BD Cytometric Bead Array (CBA) Mouse Inflammation kit (catalogue 552364, BD Bioscience) was used, according to the manufacturer’s recommendations. The kit was used for the simultaneous detection of interleukin 6 (IL-6), interleukin 10 (IL-10), interferon gamma (IFNγ), tumor necrosis factor (TNF), interleukin 12 (IL-12) and monocyte chemotactic protein (MCP-1/CCL2), in a single sample. The cytokine standards were diluted serially to construct the calibration curves, used to determine the concentrations of the cytokines. The samples were analyzed using the 13-Color CytoFLEX-S flow cytometer (Beckman-Coulter, USA). Individual cytokine concentrations were indicated by their fluorescent intensities and expressed in pg/mL, using the FCAP Array Software. The theoretical limits of detection were: 5 pg/mL for IL-6, 2.5 pg/mL for IFNγ, 7.3 pg/mL for TNF, 10.7 pg/mL for IL-12 and 17.5 pg/mL for IL-10.

### Statistical Analysis

**Figure 1:**
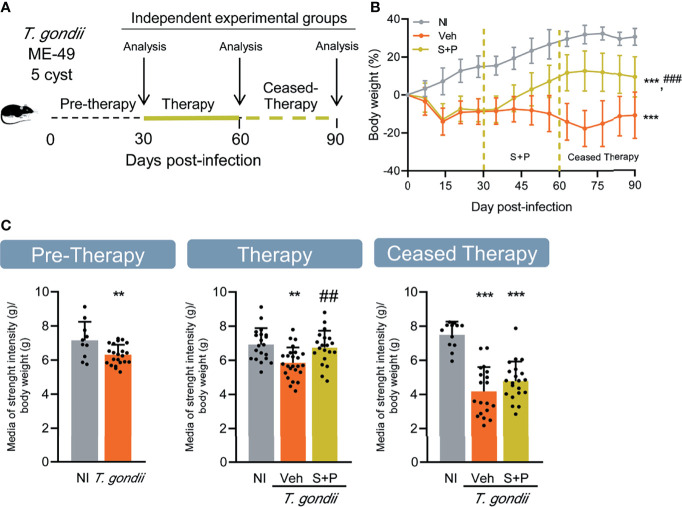
Effect of combined sulfadiazine plus pyrimethamine therapy on weight gain and muscle strength in the long-term chronic *Toxoplasma gondii* infection. **(A)** Mice were infected with 5 cysts of the ME-49 *T. gondii* strain, and after the onset of chronic long-term infection, they were treated orally with the combination of sulfadiazine plus pyrimethamine, and evaluated immediately after finishing the treatment (therapy) and 30 days after suspension (ceased therapy). **(B)** The S+P therapy led to increased gain in body weight of infected mice. **(C)** The S+P therapy reversed the loss of muscle strength in infected mice, but the effect is transient. Muscle strength values are presented as gram strength (gf)/body weight (g). Each experimental group consisted of 4-6 NI mice and 8-21 mice infected with *T. gondii*. Each circle represents an individual mouse. Data are expressed as means ± SD, and were analyzed using the Welch’s test **(B)**, and the Student´s *t*-test and the ordinary one-way ANOVA, followed by the Tukey *post hoc* test, with multiple comparisons **(C)**. **p<0.01; ***p<0.001, comparing mice infected with *T. gondii* and NI control mice. ^##^p<0.01, comparing Veh-treated and S+P-treated *T. gondii-*infected mice. ^###^p<0.001 comparing Veh-treated and S+P-treated T. gondii-infected mice.

**Figure 2:**
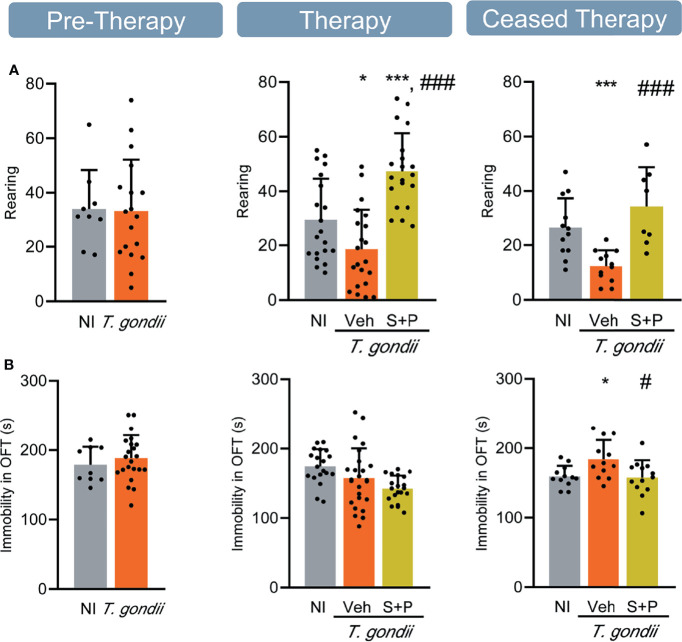
Sulfadiazine plus pyrimethamine strategy hampers locomotor and exploratory alterations in the long-term chronic *T. gondii* infection. **(A)** In long-term chronic infection the number of rearings decreased, and reversed with the S+P therapy. **(B)** The S+P therapy prevented the onset of locomotor alteration in long-term chronic *T. gondii*-infected mice. Each experimental group consisted of 4-6 NI mice and 7-14 mice infected with *T. gondii.* Each circle represents an individual mouse. Data are expressed as means ± SD, and were analyzed using the Student´s *t*-test and the ordinary one-way ANOVA followed by the Tukey *post hoc* test, with multiple comparisons **(A, B)**. *p<0.05. ***p<0.001, comparing mice infected with *T. gondii* and NI control mice. ^#^p<0.05; ^###^p<0.001 comparing Veh-treated and S+P-treated *T. gondii*-infected mice.

**Figure 3:**
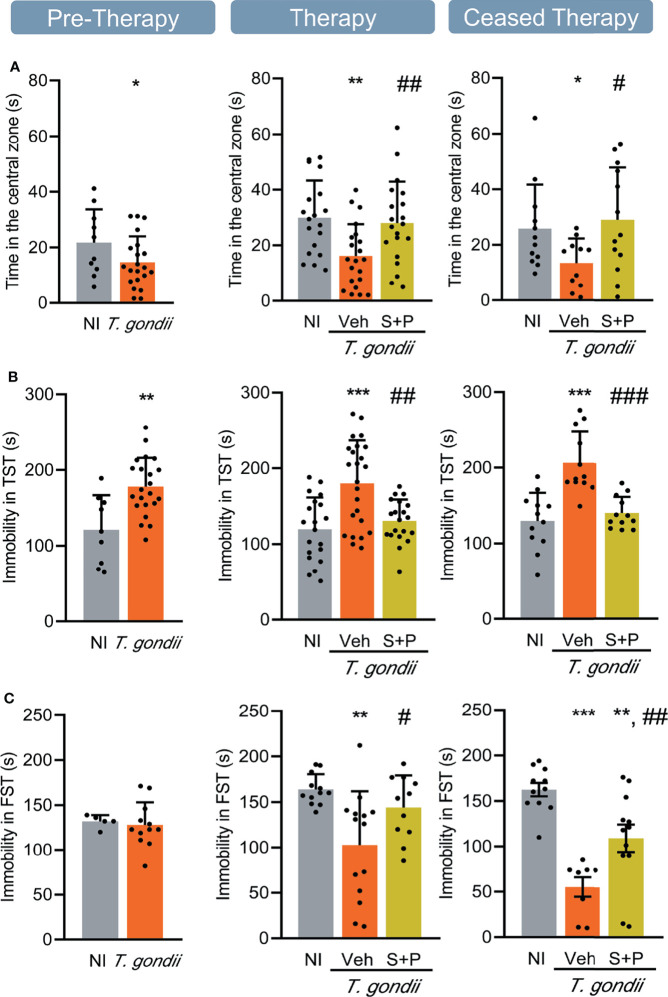
Sulfadiazine plus pyrimethamine therapy reverses behavioral changes present in the early and long-term chronic *T. gondii* infection. Chronically infected mice showed **(A)** anxiety, **(B)** depressive-like behavior, and **(C)** hyperactivity. The S+P therapy reversed these behavioral changes permanently **(A, B)** or transiently **(C)**. Each experimental group consisted of 4-6 NI mice and 8-15 mice infected with *T. gondii.* Each circle represents an individual mouse. Data are expressed as means ± SD, and were analyzed using the Student´s *t*-test and the ordinary one-way ANOVA followed by the Tukey *post hoc* test, with multiple comparisons **(A-C)**. *p<0.05; **p<0.01; ***p<0.001, comparing mice infected with *T. gondii* and NI control mice. ^#^p<0.05, ^##^p<0.01 ^###^p<0.001 comparing Veh-treated and S+P-treated *T. gondii*-infected mice.

**Figure 4:**
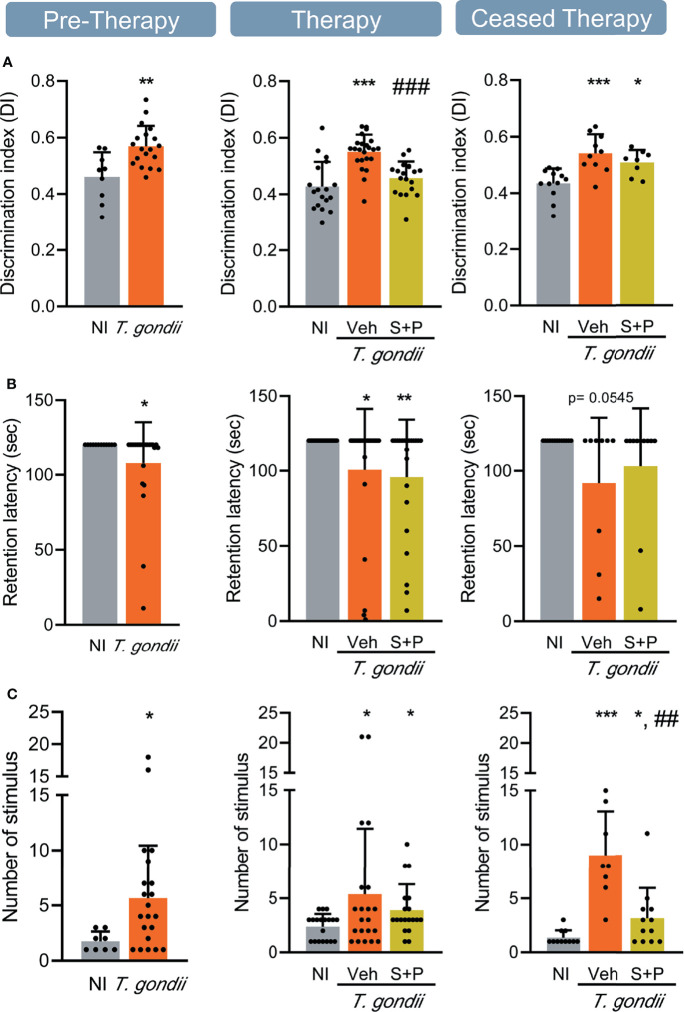
Sulfadiazine plus pyrimethamine therapy selectively reverses impairments of memory present in the early and long-term chronic *T. gondii* infection. **(A)** The effect of the S+P therapy in reversing the habituation memory alteration is transitory. **(B)** Chronically *T. gondii*-infected mice showed transient aversive memory retention impairment, or were unresponsive to the S+P therapy, and **(C)** aversive memory consolidation alterations showed partial improvement after the S+P therapy. Each experimental group consisted of 4-6 NI mice and 7-14 mice infected with *T. gondii.* Each circle represents an individual mouse. Data are expressed as means ± SD, and were analyzed using the Student´s *t*-test and the ordinary one-way ANOVA, followed by the Tukey *post hoc* test, with multiple comparisons **(A)**, the Mann-Whitney test, and the Kruskal–Wallis H test followed by *post hoc* Dunn’s multiple comparison test **(B, C)**. *p<0.05; **p<0.01; ***p<0.001, comparing mice infected with *T. gondii* and NI control mice. ^##^p<0.01; ^###^p<0.001 comparing Veh-treated and S+P-treated *T. gondii-*infected mice.

**Figure 5:**
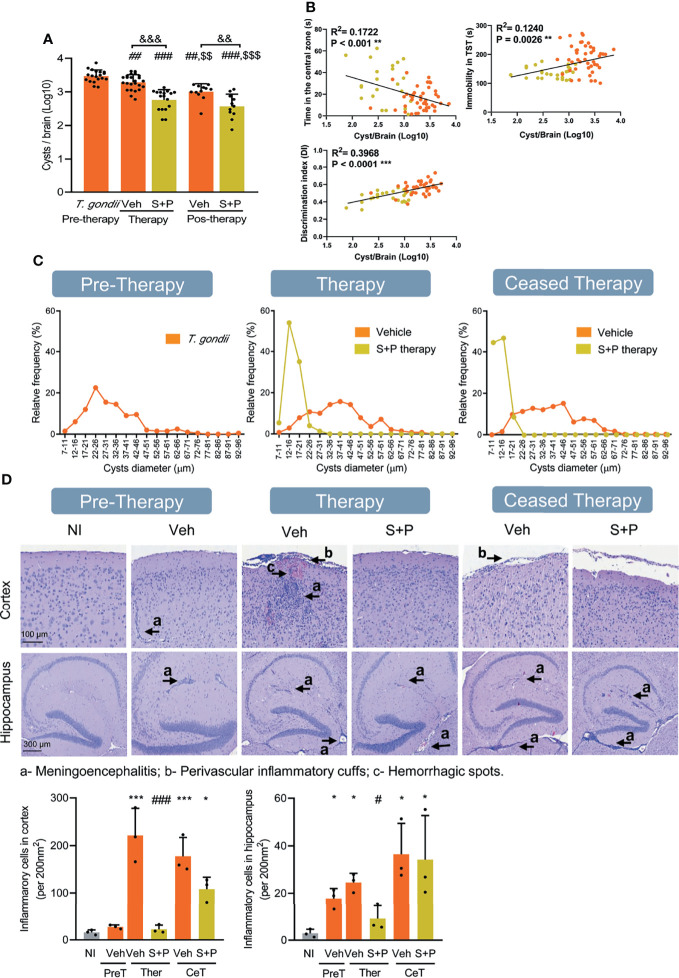
Sulfadiazine plus pyrimethamine therapy impacts the number and size of parasite cysts and histological changes in the CNS in the early and long-term chronic *T. gondii* infection. **(A)** Infected C57BL/6 mice showed reduced number of brain cysts as infection evolves from early (30 dpi, pre-therapy) to long-term (60 and 90 dpi) chronic infection. Compared with Veh-treated, S+P therapy significantly reduced the number of brain cysts, at 60 dpi (pos-therapy). The beneficial effects perssited after therapy cessation (pos-therapy). **(B)** The presence of anxiety, depressive-like behavior and habituation memory impairments was positively correlated with cyst numbers. **(C)** The sizes of brain cysts are smaller in S+P therapy mice, compared with Veh-treated mice; **(D)** In chronically infected mice, meningoencephalitis, perivascular cuffs and hemorrhagic foci are seen in the CNS areas. Bar = 100 and 300 µm. Each experimental group consisted of 8-12 mice infected with *T. gondii.* Each circle represents an individual mouse. **(A)** Data are expressed as means ± SD and were analyzed using the the Kruskal–Wallis H test followed by *post hoc* Dunn’s multiple comparison test. ^##^p<0.01; ^###^p<0.001 comparing the pre-thetrapy group whit the others group. ^$$^p<0.01; ^$$$^p<0.001 comparing the Veh-treated with others, and ^&&^p<0.01, comparing the Veh-treated whit the S+P-treated. **(B)**: Pearson’s correlation coefficient. **(D)**: Data are expressed as means ± SD, and were analyzed using the ordinary one-way ANOVA followed by the Tukey *post hoc* test, with multiple comparisons *p<0.05; ***p<0.001, comparing mice infected with *T. gondii* and NI control mice. ^#^p<0.05; ^###^p<0.001 comparing the comparing Veh-treated and S+P-treated *T. gondii-*infected mice. ^&&&^p<0.001; ^$$^p<0.01.

**Figure 6:**
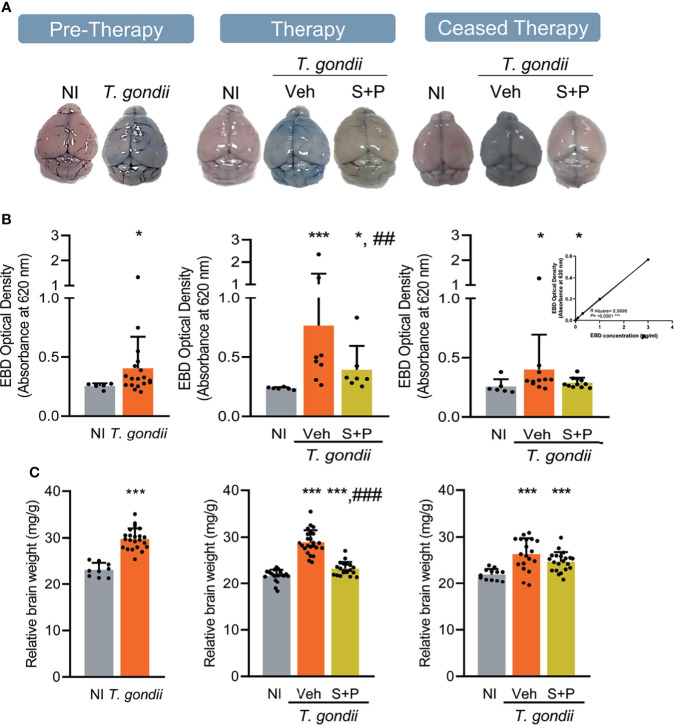
Sulfadiazine plus pyrimethamine therapy partially ameliorates the integrity of the blood-brain barrier and the cerebral edema in the early and long-term chronic *T. gondii* infection of C57BL/6 mice. **(A)** Representative brain images show EB extravasation macroscopically visible, and the S+P therapy reversed this effect. **(B)** EB concentrations increased in the brain tissues of infected mice were decreased by the S+P therapy. **(C)** Compared with NI controls, the augmented relative brain weight of infected mice, an indicative of cerebral edema, was partially ameliorated by the S+P therapy. Each experimental group consisted of 4-6 NI mice and 8-13 mice infected with *T. gondii.* Each circle represents an individual mouse. Data are expressed as means ± SD, and were analyzed using the Mann-Whitey test or the Kruskal–Wallis H test followed by *post hoc* Dunn’s multiple comparison test **(B)**, the Student´s *t*-test, the Kruskal–Wallis H test followed by *post hoc* Dunn’s multiple comparison test, and the ordinary one-way ANOVA followed by the Tukey *post hoc* test, with multiple comparisons **(C)**. *p<0.05; ***p<0.001, comparing mice infected with *T. gondii* and NI control mice, ^##^p<0.01; ^###^p<0.001 comparing the Veh-treated and the S+P-treated *T. gondii*-infected mice.

**Figure 7:**
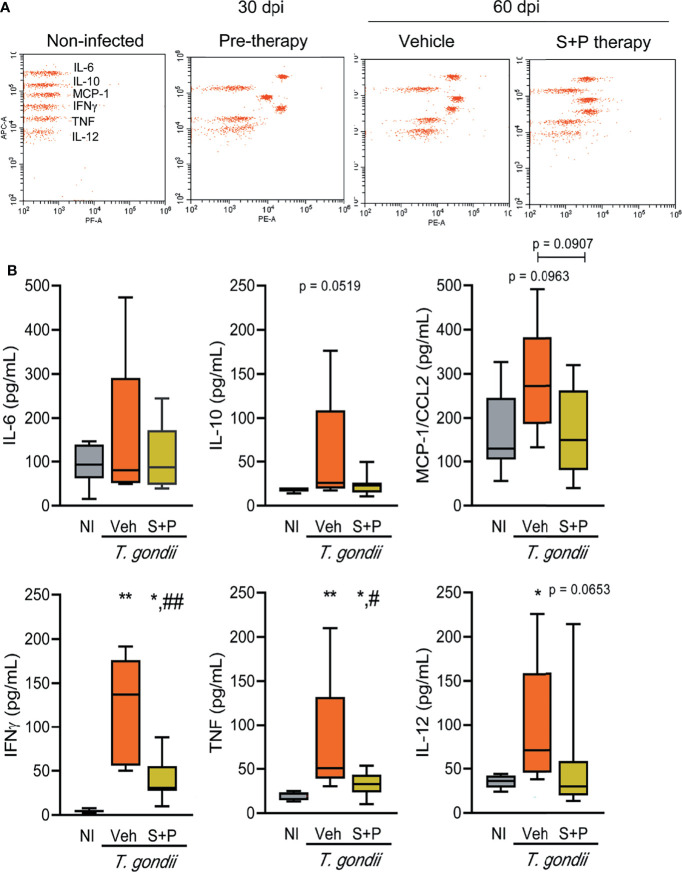
Sulfadiazine plus pyrimethamine therapy reduces the increased inflammatory cytokine serum levels in early and long-term chronic *T. gondii* infection**. (A)** The images show representative data plots of the FACS analysis of CBA of NI control, infected and therapy groups. **(B)** IL-6, IL-10, MCP-1/CCL2, IFNγ, TNF, IL-12 of the Veh-treated infected mice were elevated compared with NI controls, and the S+P therapy affected the levels of all cytokines. Each experimental group consisted of 2-3 NI mice and 2-8 mice infected with *T. gondii.* Cytokine levels are shown in box and whisker charts, with medians, and minimun and maximun values shown by vertical lines. Data were analyzed using the Kruskal–Wallis H test followed by *post hoc* Dunn’s multiple comparisons tests and the ordinary one-way ANOVA followed by the Tukey *post hoc* test, with multiple comparisons **(B)** *p<0.05; **p<0.01, comparing mice infected with *T. gondii* and NI control mice, ^#^p<0.05 ^##^p<0.01, comparing Veh-treated and S+P-treated *T. gondii*-infected mice.

To assess the normality of the data, the Shapiro-Wilk test was used. For normally distributed data formed by two groups (NI, *T. gondii*) differences were analyzed with Student’s t-test. Further, normally distributed data formed by more than two groups (NI, S+P, S+P ceased), differences between groups were analyzed using the parametric one-way ANOVA test followed by the Tukey *post hoc* test, with multiple comparisons, with a 95% confidence. Non-normally distributed data were analyzed with the non-parametric Kruskal–Wallis H test, for groups with two groups, or one-way ANOVA on ranks for analysis with more than two groups, followed by the *post hoc* Dunn’s multiple comparisons test. Data obtained using Shapiro-Wilk test are shown as **Supplementary Table S1**. Correlation was analyzed using Pearson’s correlation coefficient. Statistical tests were performed using the GraphPad Prism version 8.0. Differences were considered statistically significant when *p* < 0.05. In **Figures 1**-**6**, data are presented as scatter plot with bar graphs, each circle represents the measurement of an individual mouse, the means are shown as bars and standard deviations (SD) are shown as vertical lines above means. In **Figure 7**, cytokines levels are shown in box and whisker charts, and the minimum and maximum values are shown by vertical lines.

To visualize simultaneously and unbiasedly the most important variables involved in our analysis, we computed principal-component analysis (PCA). This statistical method allows us to identify graphically which cytokines and behaviors are more related with the number of cysts and, therefore, recognizes the values of the variables associated with the performance of the treatment groups. For PCA analyses, all studied parameters of 6 NI control mice, 6 vehicle-treated infected mice and 8 S+P-treated infected mice were included. PCA was performed on scaled normalized expression values using the built-in R function PCA() from package FactoMineR (v1.34), and the plots were generated using the ggplot2 (v3.3.3), factoextra (v1.0.7) and pca3d (v0.10.2) R packages. All R analysis was carried out using the RStudio environment (v1.4.1103) (51).

## Results

### Combined Sulfadiazine Plus Pyrimethamine Therapy Increases Weight Gain and Restores Muscle Strength in the Long-term Chronic *Toxoplasma gondii* Infection

Based on a previous kinetic study showing the establishment of the early (30 dpi) and long-term (60 and 90 dpi) chronic infection (22), female C57BL/6 mice were infected and treated with S+P therapy for 30 consecutive days (from 30 to 60 dpi), i.e., from early to late chronic infection. The independent groups were evaluated at three timepoints: (i) in the early chronic phase, pre-therapy (30 dpi); and in the late chronic infection (ii) therapy group (60 dpi) and (iii) 30 days after therapy cessation (90 dpi), as described (**Figure 1A**). A preliminary statistical analysis of the three NI control groups run concurrently with *T. gondii*-infected groups showed no difference (*p* > 0.05) between the NI controls at the three timepoints. For simplicity, and when possible, data collected from all NI mice were pooled and referred as NI in graphs and figures.

Clinical evaluation revealed loss in body weight during the acute phase of infection (up to 15 dpi). After this period, the weight loss stopped. Nevertheless, the bodyweight gained in Veh-treated infected mice remained lower than the NI controls. In the second week of S+P therapy, an increase in body weight gain in infected mice was noticed, when compared with Veh-treated mice. However, S+P-treated mice did not reach body weight comparable to the bodyweight of NI mice (**Figure 1B**). This effect was sustained one month after cessation of S+P treatment (at 90 dpi). Muscle strength progressively decreased in *T. gondii*-infected mice from the early, at 30 dpi (NI 7.149 ± 1.090 *vs T. gondii*-infected 6.305 ± 0.5918, p = 0.0078), to the long-term chronic infection, at 60 dpi (NI 6.922 ± 0.958 *vs* Veh-treated *T. gondii*-infected 5.852 ± 0.896, p = 0.0010) and 90 dpi (NI 7.492 ± 0.774 *vs* Veh-treated *T. gondii*-infected 4.174 ± 1.425, p <0.0001). At 60 dpi, compared with NI controls muscle strength loss was detected in Veh-treated, but not in S+P-treated infected mice (**Figure 1C**, Therapy, middle panel). Although therapy showed a beneficial effect on muscle strength, this effect was transient as 30 days after therapy cessation (90 dpi, Ceased Therapy) the loss of muscle strength was evident in S+P-treated mice, alike the paired Veh-treated group (**Figure 1C**, Ceased Therapy, right panel).

Behavioral and neurocognitive tests require interaction with the environment, thus firstly we evaluated exploratory and locomotor activities to check for a possible influence of loss in muscle strength detected in *T. gondii*-infected C57BL/6 mice on test performance. Motor function was assessed in the open field, broadly used to study murine models of muscle diseases as muscular dystrophy (38). The number of rearing and the immobility time were analyzed. No changes in exploratory and locomotor activities were detected in the early chronic phase (**Figure 2A**, Pre-Therapy, left panel), but a decrease in the number of rearings was detected in the long-term (60 and 90 dpi) chronic infection (**Figure 2A**, middle and right panels). Treatment with S+P hampered the onset of this alteration (**Figure 2A**, middle panel), an effect sustained one month after cessation of therapy (**Figure 2A**, right panel). At 30 and 60 dpi, infected mice showed similar time of immobility in the OFT, compared with NI matched controls (**Figure 2B**, left and middle panels). At 90 dpi, Veh-treated mice remained immobile longer than NI controls, supporting progression of behavioral changes as infection evolved from 60 to 90 dpi. Nonetheless, S+P therapy hampered the onset of this behavior (**Figure 2B**, right panel). The beneficial effects of S+P therapy were transient (restricted to Therapy group, at 60 dpi), as muscle strength, or sustained (detected in Ceased Therapy group, at 90 dpi), as weight gain, exploratory and locomotor changes. Altogether, these results indicate that *T. gondii*-infected mice are able to perform the proposed behavioral and neurocognitive tests.

### Anxiety-Like Disorder and Depressive-Like Behavior Present in the Early and Long-term Chronic *Toxoplasma gondii* Infection Are Reversed by S+P Therapy

The anxiety-like disorder was assessed using the OFT, and determined as the time spent in the central zone of the apparatus (39). Compared with NI controls, infected mice remained shorter time in the central area of the open field, at the three timepoints analyzed (**Figure 3A**). Compared with Veh-treated, S+P therapy reversed anxiety-like disorder, and such effect was maintained even after cessation of therapy (**Figure 3A**). Depressive-like behavior was revealed as enhanced time of immobility in the TST (45). When compared with NI controls, *T. gondii*-infected mice showed increased immobility time at early (30 dpi) and long-term (60 and 90 dpi) stages of the chronic infection (**Figure 3B**). This behavioral alteration was also reversed by S+P therapy (**Figure 3B**, middle panel**)**. Again, the beneficial effect of S+P was sustained one month after suspension of therapy (**Figure 3B**, right panel).

### Hyperactive Behavior in C57BL/6 Mice Chronically Infected With *Toxoplasma gondii* Is Responsive to S+P Therapy

The FST was used to assess the hyperactive behavior, detected as reduced time in immobility (46). At the early chronic phase (30 dpi), hyperactive behavior was not detected (**Figure 3C**, left panel). However, hyperactivity was present in long-term Veh-treated infected mice (60 and 90 dpi), as they remained immobile for shorter time in the FST, compared with NI controls (**Figure 3C**, middle and right panels). At 60 dpi, the combined S+P therapy hampered the onset of the hyperactive behavior (**Figure 3C**, middle panel). This effect was maintained in 58% of the S+P-treated infected mice, even 30 days after therapy discontinuation (**Figure 3C**, right panel).

### Chronically *Toxoplasma gondii*-infected C57BL/6 Mice Show Transient or Permanent Memory Impairments That Are Selectively Affected by S+P Therapy

We evaluated two types of long-term memory during chronic *T. gondii* infection. Habituation memory was assessed using the OFT, comparing the performance in the first and second days of the test (42), shown as discrimination index. In the early (30 dpi) and long-term (60 and 90 dpi) chronic phase of infection, we detected habituation memory loss (**Figure 4A**). Combined S+P therapy reversed habituation memory impairment (**Figure 4A**, middle panel); however, the beneficial effect was not sustained after cessation of therapy (**Figure 4A**, right panel). Aversive memory was assessed by the passive avoidance task (48). At 30 and 60 dpi, ME-49-infected female C57BL/6 mice showed impairment of the aversive memory retention (**Figure 4B**, left and middle panels). At 60dpi, S+P therapy did not impact memory retention (**Figure 4B**, middle panel). However, in the ceased therapy group (90 dpi), 67% of the Veh-treated and 82% of the S+P-treated infected mice presented intact aversive memory (**Figure 4B**, right panel), suggesting a transient impairment of this memory and a slight beneficial effect of the S+P therapy. In addition, changes in aversive memory consolidation, shown as an increase in the number of stimuli needed to acquire memory, was detected in infected mice throughout infection, compared with paired NI controls (**Figure 4C**). At 60 dpi, no impact of therapy was detected (**Figure 4C**, middle panel). Long-term analysis after therapy cessation (at 90 dpi) showed that S+P treatment reduced the number of stimuli required for aversive memory consolidation, compared with Veh administration (**Figure 4C**, right panel), also supporting beneficial effects of S+P therapy.

### Therapeutic Intervention With S+P Reduces the Number of Brain Cysts

Next, we challenged the relation of *T. gondii* brain cysts with behavioral and neurocognitive changes. As infection evolved from the early (30 dpi) to the long-term (60 and 90 dpi) chronic infection, a gradual and significant reduction in the number of cysts was detected in the encephalon (**Figure 5A**), indicating a continuous infection control. At 60 dpi, fewer cysts were seen in the encephalon of mice of the S+P therapy group, compared with the Veh-treated group. This effect persisted after cessation of therapy, at 90 dpi (**Figure 5A**). A significant correlation was observed between the number of cysts in the CNS and anxiety-like disorder (*p* < 0.001), depressive-like behavior (*p* = 0.0026), and habituation memory loss (*p* < 0.0001). Further, these data reinforced the beneficial role of the S+P therapy leading to reduction in the number of cysts in the CNS concurrent with improvement of behavioral alterations (**Figure 5B**). Further, the cysts were classified according to diameters into 18 classes and expressed as relative frequency of each class (**Figure 5C**). In comparison with the prominent frequency of cysts in the size class <37-41nm in the early (30 dpi) chronic infection, we observed an increase in the frequency of cysts in the class >42-46 µm as infection evolves to the long-term (60 and 90 dpi) chronic infection (**Figure 5C**). The sizes of the cysts were smaller in S+P therapy grouup in comparison with Veh administration, showing a higher relative frequency of cysts <12-26 µm, and this feature persisted after therapy cessation (**Figure 5C**), reinforcing the effect of therapy on control of brain cyst load.

### Combined S+P Therapy Reduces the Severity of Neuropathological Alterations in Chronically *Toxoplasma gondii*-infected C57BL/6 Mice

In the early (30 dpi) and long-term (60 and 90 dpi) chronic infection mice showed meningoencephalitis and perivascular inflammatory infiltrates, mainly composed of mononuclear cells, with rare hemorrhagic events (**Figure 5D**). Compared with Veh administration, less intense meningitis and reduced number of hemorrhagic foci and mononuclear inflammatory cells infiltrating the CNS cortex and hippocampus were seen in S+P-treated mice, at 60 dpi (**Figure 5D**). These beneficial effects were not observed after therapy discontinuation (**Figure 5D**).

Next, we used EB dye to assess BBB integrity (49, 50). Compared with NI controls, the BBB of infected mice was disrupted in the early (30 dpi) and long-term (60 and 90 dpi) chronic phase of the infection. The CNS tissue tonality turned blue due to extravasation of the EB dye into the tissue, which can be observed macroscopically (**Figure 6A**). When compared to age-matched NI controls, increased EB concentrations were detected in the brain of infected mice, at all analyzed endpoints (**Figure 6B**). Lower BBB disruption was detected macroscopically (**Figure 6A**) and colorimetrically in S+P-treated infected mice (**Figure 6B**). Indeed, BBB integrity was observed in 80% (12/15) of S+P-treated mice. Even one month after therapy discontinuation, this beneficial effect was observed in 91% (10/11) of the S+P-treated infected mice (**Figure 6B**). In addition, compared to age-matched NI controls, non-treated or Veh-treated infected mice showed an important increase in the relative brain weight (**Figure 6C**). At 60 dpi, in comparison with Veh administration the relative brain weight was reduced in S+P treated mice. However, this beneficial effect was not observed 30 days after therapy cessation (at 90 dpi), as Veh- and S+P-treated mice presented increased relative weight of the brain, in comparison with NI controls (**Figure 6C**). Thus, indicating that the S+P therapy beneficial effect on the cerebral edema present in chronically *T. gondii*-infected female C57BL/6 mice was transient.

### Elevated Systemic Levels of Cytokines in the Early and Long-term Chronic *Toxoplasma gondii* Infection Are Decreased by S+P Therapy

Lastly, we assessed the systemic immune response evaluating the serum levels of cytokines (IL-6, IL-10, MCP-1/CCL2, IFNγ, TNF, IL-12) in NI controls and *T. gondii*-infected mice. At 30 dpi (pre-therapy) and 60 dpi, among the Veh-treated infected mice IL-6, IL-10, MCP-1/CCL2, IFNγ, TNF, IL-12 levels in serum were elevated, compared with NI controls (**Figures 7A, B** and **Table 1**). S+P therapy affected the levels of all cytokines (**Figures 7A, B**); however, significant reduction was detected only in IFNγ and TNF serum levels (**Figures 7A, B** and **Table 1**). At 90 dpi, the cytokine levels in serum of the Veh- and the S+P-treated infected mice tended to decrease, and cytokine levels were alike those found in serum of NI controls, except for IFNγ, MCP-1/CCL2 and IL-12 levels that remained elevated (**Supplementary Figure S2**, **Table 1**).

**Table 1:** Cytokine levels in serum of *Toxoplasma gondii*-infected mice.

		Pre-therapy		Therapy				Ceased Therapy			
	NI Control	Veh		Veh		S+P		Veh		S+P	
Cytokine	Mean ± SEM	Mean ± SEM	p- value	Mean ± SEM	p- value	Mean ± SEM	p- value	Mean ± SEM	p- value	Mean ± SEM	p- value
IL-6	93.87 ± 19.36	225.10 ± 41.96	**0.015**	153.00 ± 80.70	0.456	107.90 ± 26.23	0.693	97.04 ± 57.61	•0.857	90.87 ± 16.88	•0.857
IL-10	19.91 ± 1.68	26.00 ± 5.68	0.262	56.47 ± 30.20	•0.052	23.67 ± 4.18	•0.282	16.01 ± 3.22	•0.222	19.09 ± 0.10	•0.5
MCP-1/CCL2	164.5 ± 34.89	360.00 ± 33.68	**0.003**	281.9 ± 58.40	0.096	164.1 ± 34.92	0.995	319.6 ± 40.40	**0.012**	207.20 ± 54.82	0.225
IFNγ	4.667 ± 0.69	180.20 ± 13.96	**<0.0001**	120.2 ± 27.68	**0.0005**	39.84 ± 8.51	**0.002**	110.30 ± 79.39	**0.017**	70.69 ± 20.86	**0.0008**
TNF	19.79 ± 1.74	65.74 ± 14.33	**0.003**	78.56 ± 33.08	**•0.003**	32.45 ± 4.88	**•0.040**	28.32 ± 10.59	0.177	38.04 ± 2.39	**0.001**
IL-12	35.09 ± 2.85	43.56 ± 11.24	0.413	95.83 ± 33.71	**•0.018**	55.05 ± 23.42	•0.779	18.62 ± 5.68	•0.111	43.54 ± 0.49	•0.222

•p-values calculated using the Mann-Whitney test. Statistically significant p-value < 0.05 (bold); infected group compared to NI control group.

### Systematization of Data

**Table 2** shows a summary of the obtained data, independently rated from 0 to 4 for each analyzed parameter. Thus, two independent observers considered a feature (eg, levels of cytokine) or performance in a test in NI controls as absence of alteration (0 = absent). Alterations detected in *T. gondii*-infected mice were graded from mild to severe (1= mild; 2= moderate; 3=severe). Prior the analysis of data and to establish a fair score system, the possibility of exacerbation of a feature due to S+P therapy was also considered (4= aggravation). In the early (30 dpi) and long-term (60 and 90 dpi) chronic *T. gondii* infection, behavioral and neurocognitive changes were detected. Importantly, the S+P administration to infected mice in the early chronic phase brought selective beneficial effects that were transient or sustained 30 days after cessation of therapy. No aggravation of any detected abnormality was observed in the S+P-treated groups, compared with their matched Veh-treated groups. Indeed, the S+P therapy resolved locomotor alterations, anxiety-like disorder, and depressive-like behavior, partially or transiently solved hyperactivity and habituation memory loss, but presented no effect on aversive memory changes. The beneficial effects of the S+P therapy were paralleled by lower brain cyst load, and less intense neuroinflammation, BBB disruption and brain edema. Lastly, the favorable impact of the S+P therapy was associated with a decline of the pro-inflammatory Th1 cytokines IFNγ and TNF levels in serum. Altogether, our data support that although acting selectively or transiently on neurocognitive impairments, the S+P therapy resolved locomotor and behavioral (anxiety and depression) alterations.

**Table 2:** Effectiveness of sulfadiazine plus pyrimethamine therapy in long-term chronic *T. gondii* infection.

		Pre-therapy	Therapy		Ceased Therapy	
Parameter	NI Control	Veh	Veh	S+P	Veh	S+P
**Weight loss**	0	2	1	0	1	0
***Neuromuscular function***						
Muscle strength	0	1	1	0	2	2
***Motor function***						
Rearing	0	0	1	0	1	0
Immobility time (OFT)	0	0	0	0	1	0
***Behavioral changes***						
Anxiety	0	1	1	0	1	0
Depression	0	1	1	0	1	0
Hyperactivity	0	0	1	0	3	1
***Cognitive impairments***						
Habituation memory	0	1	1	0	1	1
Aversive memory retention	0	1	1	1	0	0
Aversive memory consolidation	0	3	3	2	3	1
***Brain cyst***						
Number	0	3	3	2	2	1
Size	0	2	3	1	3	1
***Neuropathological alterations***						
Meningoencephalitis	0	2	3	1	3	2
Perivascular infiltrates	0	2	3	1	3	2
BBB integrity	0	2	3	1	2	1
***Cytokine regulation***						
IL-6	0	3	2	1	0	0
IL-10	0	1	2	1	0	0
MCP-1/CCL2	0	3	2	1	3	2
IFNγ	0	3	3	1	3	2
TNF	0	3	3	2	1	2
IL-12	0	1	3	2	0	1
**Total score**	**0**	**35**	**41**	**17**	**34**	**19**

The changes and the effect of the therapy were rated from 0 to 4, being 0 = absent; 1= mild; 2= moderate; 3=severe; 4= aggravation. Bold values: Total score of effectiveness of S+P treatment.

**Figure 8:**
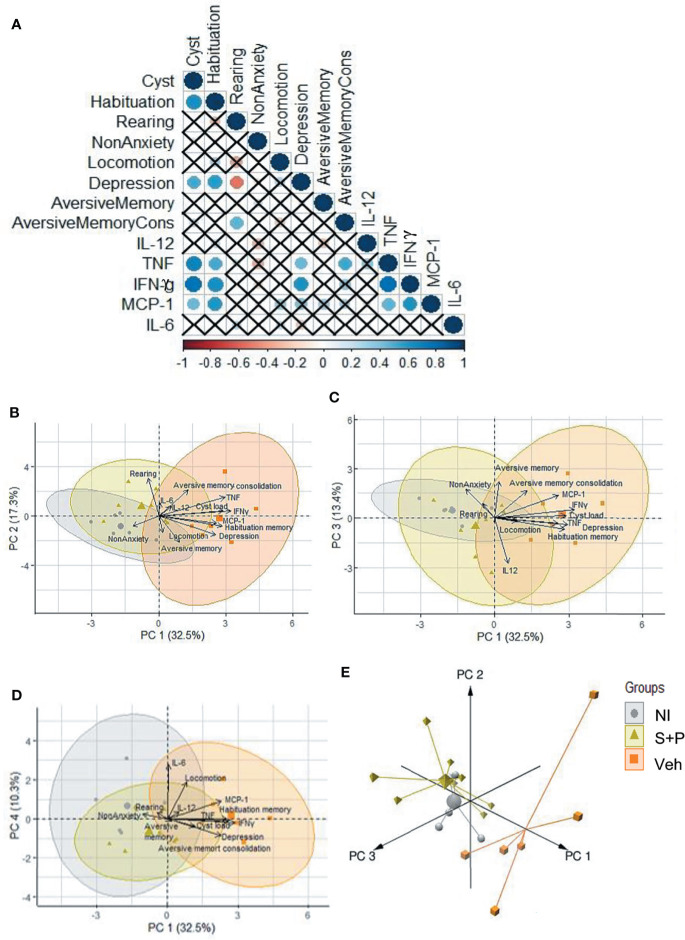
Relationship between brain cyst load, cytokines and behavioral and neurocognitive changes. **(A)** Significant correlation is represented by a circle, the color indicates the strength of this correlation. The “x” indicates the absence of a significant correlation between the variables. **(B-D)** The four first principal components explain 73.5% of the variance, and the 2D projection of **(B)** PC1-PC2, **(C)** PC1-PC3 and **(D)** PC1-PC4 indicated the association between brain cyst load, serum cytokine levels and behavioral and neurocognitive abnormalities. **(E)** The 3D projections, with the three first principal showed that the NI control and the S+P-treated clusters showed a similar behavior, and demonstrated the differences between the Veh-treated and NI control cluster and the S+P-treated and the Veh-treated cluster.

A correlation analysis clearly revealed significant correlation between IFNγ, TNF and MCP-1/CCL2 levels in serum with brain cyst load. Likewise, the correlation showed impairment of habituation memory and depression behavior associated with brain cyst load and elevated systemic levels of IFNγ and TNF. The CC-chemokine MCP1/CCL2 showed a relationship with impairment of habituation memory. Finally, a weak association between TNF levels and impairment of consolidation of aversive memory was also observed (**Figure 8A**). Further, to visualize the similarities between groups and identify putative clusters of differentiation, we performed a PCA using data of behavioral and neurocognitive abnormalities and proinflammatory cytokine levels in serum, comparing NI controls, the Veh-treated and the S+P-treated infected mice. The four first principal components were used in order to explain 73.5% of the variance. The 2D projection of the samples using the two first principal components, which explain 49.81% of the variance, confirmed the association between brain cyst load, serum levels of the cytokines IFNγ, TNF and MCP-1/CCL2 and behavioral and neurocognitive abnormalities (**Figure 8B**). PC3 and PC4 showed similar behavior as shown in PC1 and PC2 (**Figures 8C, D**). We found that 3D projections, with the three first principal components explaining the 63.25%, emphasized the differences between the three groups, distinguishing the Veh-treated from the NI control cluster and the S+P-treated from the Veh-treated cluster. Moreover, the NI control and the S+P-treated clusters presented a similar behavior (**Figure 8E**). In summary, PCA indicates that brain cyst load and elevated systemic proinflammatory cytokine levels are associated with behavioral/neurocognitive changes in the Veh-treated infected mice. Conversely, low levels of cytokines and absence or lower brain cyst load are associated with the absence of behavioral/neurocognitive alterations in the NI controls and resolution/amelioration of these changes in the S+P-treated mice. Altogether, these data support that the S+P therapy adds significant advantage to the cyst control already observed in ME-49-infected female C57BL/6 mice as infection evolves from the early to the long-term chronic infection, thus impacting behavioral and neurocognitive changes.

## Discussion

In humans, depression (15, 52), anxiety, hyperactivity (53) and alteration of neurocognitive functioning (16, 53) have been related to infection by the *T. gondii*. Here, some of these behavioral and neurocognitive changes were replicated in ME-49-infected C57BL/6 mice. In the present study, to shed light on the mechanisms underlying behavioral alterations in *T. gondii* infection, firstly we described that in the early and long-term chronic infection female C57BL/6 mice showed locomotor disorders, behavioral alterations (anxiety-like disorder, depressive-like behavior, and hyperactivity) and neurocognitive impairments (habituation and, partially, aversive memory loss). Mostly, these abnormalities were associated with brain cyst load, neuroinflammation, BBB disruption, and systemic inflammatory profile. Although this model brain cyst load is reduced as infection evolves from early to long-term chronic phase, it was not associated with improvement of behavioral and immune-related abnormalities. Depending on the studied feature, a selective (total, partial or transient) beneficial effect of the S+P therapy was detected on behavioral and neurocognitive alterations, which were paralleled by reduction of brain cyst load and neuroinflammation, partial amelioration of the BBB disruption and decrease in the systemic inflammatory cytokine levels. Correlation analysis revealed association between IFNγ, TNF and MCP-1/CCL2 serum levels, brain cyst load and behavioral and neurocognitive alterations. Moreover, PCA 2D and 3D projections clearly highlighted the existence of three distinct clusters (NI controls, Veh-treated and S+P-treated) and, particularly, distinguished the clusters representing the groups of the Veh- and the S+P-treated infected mice.

*T. gondii*-infected mice showed body weight loss during the acute phase of infection (15 dpi), which ceased in the early and long-term chronic infection. This dynamic of weight loss has been widely documented in murine models (9, 25, 54–56). Here, mice treated with the S+P in the early chronic phase showed increase of body weight, compared to themselves before therapy and Veh-treated infected controls. The S+P treatment in the acute phase of infection of C57BL/6 mice with the Fukaya (type II) strain hampered weight loss, supporting that the S+P therapy controlled parasite dissemination and, as consequence, suppressed body weight loss (56). Decreased muscle strength, another common feature of murine *T. gondii* infection (9, 17, 55), was replicated in ME-49-infected female C57BL/6 mice (22). S monotherapy administered to chronically ME-49-infected Swiss Webster mice did not restore muscle strength (9). Presently, we showed that even though it is transient, the S+P therapy restored neuromuscular strength, supporting the need of the combined therapy.

In the early (30 dpi) and long-term (60 and 90 dpi) chronic infection, alterations in locomotor and exploratory activities, behavioral changes, and neurocognitive impairments were concurrently recorded in the ME-49-infected female C57BL/6 mice. Decreased numbers of rearing (17), anxiety (18–22, 34), hyperactivity (20, 22–25), impaired spatial memory (28) and alteration in aversive memory consolidation (26, 27) have been reported independently in different studies using murine models of *T. gondii* infection. However, discordant findings have also been reported in *T. gondii*-infected mice, as locomotor alterations with increased number of rearings (19) or the absence of any type of locomotor alteration (21, 27), the absence of anxiety-like disorder (17, 25, 27) and even decreased general anxiety (34). These dissonant results may be attributed to differences between the host (murine lineages and gender), the parasite (*T. gondii* strains, infective stages and inoculum) and settings to assess behavioral changes (methodologies, environmental conditions, timepoints of evaluation after infection) (57). Therefore, the here used experimental model using C57BL/6 mice infected with low inoculum (5 cysts) of the ME-49 strain simultaneously replicates relevant behavioral and neurocognitive changes detected in the long-term human toxoplasmosis (15, 16), and therefore is appropriate to challenge the effects of the intrinsic immune response controlling brain cysts (22) and the effects of the S+P therapy on these changes.

The effects of the intrinsic brain cyst control as infection evolves from early to long-term chronic infection in ME-49-infected female C57BL/6 mice (22) was replicated here, contrasting with the increase in cyst load in ME-49-infected male B6CBAF1/J mice (34). A relation between parasite load and severity of behavioral changes, such as loss of predator’s fear, exploratory behavior and anxiety has been observed (34). In C57BL/6J mice infected with genetically modified tachyzoites of the ME-49 strain, only mice with brain cysts but not the ones devoid of cysts in the CNS exhibited behavioral changes (25), supporting that parasite cysts in the CNS are crucial for these abnormalities. Conversely, behavioral changes, as loss of aversion to the predator’s urine, were shown to be independent of the presence of the parasite in the CNS (58), implicating other factors in these alterations. Here, our data show that reduction in brain cyst number as infection evolves from the early to late chronic infection was not enough to improve behavioral and neurocognitive changes. Thus, we proposed the use of the S+P therapy to add advantage to the immune-mediated cyst control (10–12), and with the obtained effect of reduced number of cysts in the CNS, we challenged the effects on behavioral and neurocognitive change. We observed a positive correlation between the number of cysts in the CNS and behavioral and neurocognitive alterations. The reduction of the number of brain cyst with the S+P therapy was related to transient amelioration of muscle strength loss, hyperactivity and habituation memory, partial amelioration of BBB disruption and of impaired aversive memory consolidation, and no further detection of anxiety and depressive-like behavior. In addition, the S+P scheme drastically reduced the number of brain cysts and the presence of “large cysts”, and this beneficial effect persisted after ceased therapy. These effects of S+P therapy require replication in other experimental models. Also, the long-term biological implications for the reduction of the number of cysts and the presence of small, but not large, cysts after S+P therapy need to be explored. A previous study interpreted the presence of these “small cysts” as the formation of newly seeded cysts, which may represent a continuous low-level reactivation in the CNS (59). Despite that, alterations of retention and consolidation of aversive memory are still present. Notably, there was a positive correlation between the number of cysts and anxiety, depressive-like behavior, and habituation memory impairment. Beneficial effects of the combined S+P therapy have been shown previously. Children and infants, who were born with severe involvement of the CNS because of congenital toxoplasmosis and treated for one year with the S+P plus folinic acid presented normal development and preserved intellectual function (60). In a randomized study carried out with AIDS patients with toxoplasmic encephalitis, the S+P therapy offered a better primary outcome in terms of mortality and brain herniation compared to trimethoprim-sulfamethoxazole (TMP-SMX) (61). Crucially, S+P-treated *T. gondii*-seropositive AIDS patients experienced complete resolution or partial improvement of toxoplasmic encephalitis, which, however, relapsed weeks after therapy discontinuation (62). Tests of effectiveness of the S+P therapy in murine models explored survival (63–65), tissue damage (66) and parasite load in distinct organs (64, 65, 67). Combined S+P administered to acutely ME-49-infected CF1 mice reduced brain cysts (65). Nevertheless, effectiveness of the S+P therapy on behavioral and neurocognitive functions in chronic infection has been neglected, despite alternative treatments have been tested. Chronically ME-49-infected BALB/c mice treated with rosuvastatin, which reduces the burden of brain cysts and attenuate neuroinflammation, showed reversal of anxiety and impairment of memory of recognition of new objects (21). Acutely VEG type III strain-infected Swiss mice treated with resveratrol plus TMP-SMX showed reduced number of brain cysts and amelioration of anxiety and aversive memory (27). Interestingly, in infected C57BL/6J mice, guanabenz, an anti-hypertensive drug effectively reduced hyperactivity, neuroinflammation and perivascular cuff, independently of the brain cyst load (23). Similarly, in chronically Prugniaud strain-infected BALB/cJ mice guanabenz reversed hyperactivity without a decrease in the number of brain cysts (23). Overall, improvement in behavioral and neurocognitive changes after therapy, with or without decline in brain cyst load, supports additional biological mechanisms underlying these alterations in *T. gondii* infection.

Here, we showed that in the long-term chronic *T. gondii* infection the S+P therapy, compared with vehicle treatment, reduced brain cyst load and ameliorated meningoencephalitis and inflammatory foci and hemorrhagic spots in the parenchyma. In a model of reactivated toxoplasmosis in ME-49-infected interferon regulatory factor 8-deficient mice, the S+P therapy hampered the onset of toxoplasmic encephalitis and controls brain parasitism (68). In another model, the S monotherapy administered from the acute to early chronic phase partially improved microvascular damage and reduced parasite load in the brain (69). Further, monotherapy with S or P in ME-49-infected CBA/Ca mice did not alter neuroinflammation (66). In chronically ME-49-infected Swiss Webster mice, the S monotherapy was not effective for treating neuropathological signs, despite the reduced number of cysts (9). Therefore, our data show for the first time that combining the S+P therapy was more effective than the S or the P monotherapy to promote parasite control and reduce neuroinflammation in long-term chronic *T. gondii* infection is quite relevant.

BBB integrity was impaired during the early (30 dpi) and long-term (60 and 90 dpi) chronic *T. gondii*-infection of female C57BL/6 mice, as shown previously (22). Monotherapy with S administered to Swiss Webster mice from the acute to chronic phase partially restored BBB integrity in 27% (69). Our data show that the S+P therapy restored efficiently BBB integrity, reducing EB dye leakage into the CNS in 80% of the infected mice, reinforcing the efficacy of combined therapy.

Our mouse model of early and long-term chronic infection presents a Th1 immune response profile, which is crucial to establish an efficient antiparasitic response (8). More, chronically ME-49-infected infected mice exhibited high systemic levels of the inflammatory cytokines IL-12, IL-6, TNF, IFNγ and MCP-1/CCL2, as previously reported (22). Importantly, brain cyst load was correlated with IFNγ, TNF and MCP-1/CCL2 serum levels. The S+P therapy resulted in reduction of inflammatory cytokine concentrations in serum, particularly decreasing the Th1 cytokines IFNγ and TNF levels. We noticed that although IFNγ and MCP-1/CCL2 levels were notably reduced, they remained elevated when compared with NI controls. In murine toxoplasmosis, one of the main functions of IFNγ is the induction of TNF, which is produced by macrophages, microglial cells and astrocytes in the CNS, playing a role in parasite control, thus enabling mice survival. Indeed, anti-TNF antibody aggravates toxoplasmic encephalitis in mice (70). In addition, the levels of the regulatory cytokine IL-10 increased in pre-therapy (30 dpi) and in the Veh-treated (60 dpi) mice, and the maximum values were reduced after the S+P therapy. In ME-49-infected C57BL/6 mice, short-term S monotherapy reduced IL-12, IL-10 and IFNγ levels (71). Here, we showed that 30 days after the S+P cessation of the therapy, the regulatory effect was sustained, except for IFNγ and TNF levels that remained upregulated, compared with NI controls, at this timepoint. Altogether, these data indicate that the effects of the S+P therapy, reducing neuroinflammation and systemic cytokines levels, were partially sustained (summarized in **Table 2**). Furthermore, multivariate and principal component analyses support the relationship between behavioral and neurocognitive alterations and elevated systemic cytokine levels. Thus, our results point toward systemic Th1 cytokines as key players to be further challenged as trait-related biomarker associated with efficacy of treatments for behavioral and neurocognitive changes in chronic *T. gondii* infection.

We have reported previously that in ME-49-infected female C57BL/6 mice, upregulation of intracerebral levels of TNF, MIP1α/CCL3, MIP1β/CCL4, RANTES/CCL5 and MCP-1/CCL2 precedes the onset of neuroinflammation and persisted after the establishment of behavioral alterations anxiety, depressive-like behavior and hyperactivity (22). In ME-49-infected male B6CBAF1/J mice, behavioral changes occur in a scenario of downregulation of physiological signaling (amine ligand-binding and serotonin receptors) and upregulation of inflammatory signaling pathways in the CNS (34). Brain inflammation may present a causal effect in the development of psychiatric disorders and cognition loss (29). In humans, peripheral immunostimulation leads to impaired spatial memory (72). Further, systemic administration of IFNα can induce anxiety and depression in hepatitis virus-infected patients (33). Factors related to inflammation, such as peripheral pro-inflammatory cytokines, have been the subject of research as biomarkers in psychiatric disorders (30). Chronic *T. gondii* infection induces a wide variety of behavioral and neurocognitive changes, which may depend on the experimental models and protocols used for evaluation, as already mentioned (57), but having in common the presence of neuroinflammation, a feature of *T. gondii* infection (20–23, 25, 27, 34, 62). Thus, it is reasonable that the behavioral and neurocognitive changes reflect the process of persistence of cysts in the brain tissue, but also in peripheral tissues, which can continuously stimulate the upregulation of systemic pro-inflammatory cytokine levels, and may increase BBB permeability and leak into the CNS, sustaining neuroinflammation, an idea supported by the profile of responses to therapy showed in the present study. Indeed, PCA supports the relation of systemic cytokine levels with behavioral and neurocognitive changes, reinforcing those distinct clusters reveal the beneficial effects of the S+P therapy. Thus, our study replicates in ME-49-infected C57BL/6 mice some aspects of behavioral and neurocognitive alterations reported in humans during chronic *T. gondii* infection (15, 16, 52, 53). Moreover, here we show a possible benefit of using etiological therapy in the chronic phase of toxoplasmosis to impact behavioral and cognitive changes. Importantly, one should be aware that experimental models offer limited translational potential in relation to human diseases (73). The model used here is more susceptible to infection by *T. gondii*, so it develops a greater amount of cysts and neuroinflammation, than resistant models such as BALB/c (H-2^d^) and C3H/HeN (H-2^k^), supporting difference in susceptibility to infection with this parasite, like other parasites (74). On the other hand, type II strains, such as the ME-49, are strains with a reduced virulence, which allows the study of the acute and chronic phase of the infection. Indeed, the C57BL/6 model is widely used to study chronic toxoplasmosis and behavioral changes in rodents (17, 23, 25).

Regardless of the limitation of our results for the lack of known status of brain cytokines and other neuromediators and neurotransmitters in the S+P-treated infected mice, the favorable and, in some cases, sustained action of this therapeutic scheme on cyst control, systemic cytokine levels, and behavioral and neurocognitive changes reinforce that chronically *T. gondii*-infected patients may benefit from the combined S+P therapy and have a better quality of life.

## Data Availability Statement

The original contributions presented in the study are included in the article/**Supplementary Material**. Further inquiries can be directed to the corresponding author.

## Ethics Statement

The experimental procedures were performed in accordance with the recommendations of the Guide for the Care and Use of Laboratory Animals of the National Council for Animal Experimentation. The Animal Use Ethics Committee of Oswaldo Cruz Institute/Fiocruz approved all procedures performed in this study (license L014/2018).

## Author Contributions

Conceived and designed the experiments: LCB, JL-V. Performed the experiments: LCB, ASP, DG, JL-V. Analyzed the data: LCB, LH-V, DG, JL-V. Wrote the paper: LB, JL-V. Discussed data and revised the manuscript: LCB, AS, LH-V, JM, NS, JL-V. All authors contributed to the article and approved the submitted version.

## Funding

This work was supported by grants from Fundação Carlos Chagas Filho de Amparo à Pesquisa do Estado do Rio de Janeiro/FAPERJ (E-26/210.190/2018) and the Brazilian Research Council/CNPq (BPP-304474/2015-0, BPP 306037/2019-0, INCTV, National Institute for Science and Technology for Vaccines), Grant PAEF2-IOC/Fiocruz., JM, NS, and JL-V are research fellows of the Brazilian Research Council/CNPq. JL-V is recognized with the grant Scientist of the State of Rio de Janeiro/FAPERJ (E-26/202.572/2019). This study was supported in part by the “Coordenação de Aperfeiçoamento de Pessoal de Nível Superior do Brasil” (CAPES) - Finance Code 001. The funders had no role in study design, data collection and analysis, decision to publish, or preparation of the manuscript.

## Conflict of Interest

The authors declare that the research was conducted in the absence of any commercial or financial relationships that could be construed as a potential conflict of interest.

## Publisher’s Note

All claims expressed in this article are solely those of the authors and do not necessarily represent those of their affiliated organizations, or those of the publisher, the editors and the reviewers. Any product that may be evaluated in this article, or claim that may be made by its manufacturer, is not guaranteed or endorsed by the publisher.

## Supplementary Material

Supplementary Figure 1 Flow chart showing the experimental protocol with the experimental *n* in 2 (pre-therapy and therapy group) or 1 (ceased therapy group) independent experiments and survival.

Supplementary Figure 2 Cytokine serum levels pre-Therapy and after Ceased Sulfadiazine plus pyrimethamine therapy. **(A)** Concnetrations of IL-6, IL-10, MCP-1/CCL2, IFNγ, TNF, IL-12 in serum of NI control mice and *T. gondii*-infected mice, at 30 dpi. **(B)** Concnetrations of IL-6, IL-10, MCP-1/CCL2, IFNγ, TNF, IL-12 in serum of NI control mice and Vehicle-treated and S+P-treated *T. gondii*-infected mice, at 90 dpi. Each experimental group consisted of 2-3 NI mice and 2-8 mice infected with *T. gondii.* Cytokine levels are shown in box and whisker charts, with medians, and minimun and maximun values shown by vertical lines. Data were analyzed using the Kruskal–Wallis H test followed by *post hoc* Dunn’s multiple comparisons tests and the ordinary one-way ANOVA followed by the Tukey *post hoc* test, with multiple comparisons **(A**, **B)** *p<0.05; **p<0.01; ***p<0.001comparing mice infected with *T. gondii* and NI control mice.
