# Supplementary figures and images for: Sulfadiazine Plus Pyrimethamine Therapy Reversed Multiple Behavioral and Neurocognitive Changes in Long-Term Chronic Toxoplasmosis by Reducing Brain Cyst Load and Inflammation-Related Alterations
Source: Front Immunol. 2022 Apr 27;13:822567. doi: 10.3389/fimmu.2022.822567 (PMC9091718; doi:10.3389/fimmu.2022.822567)

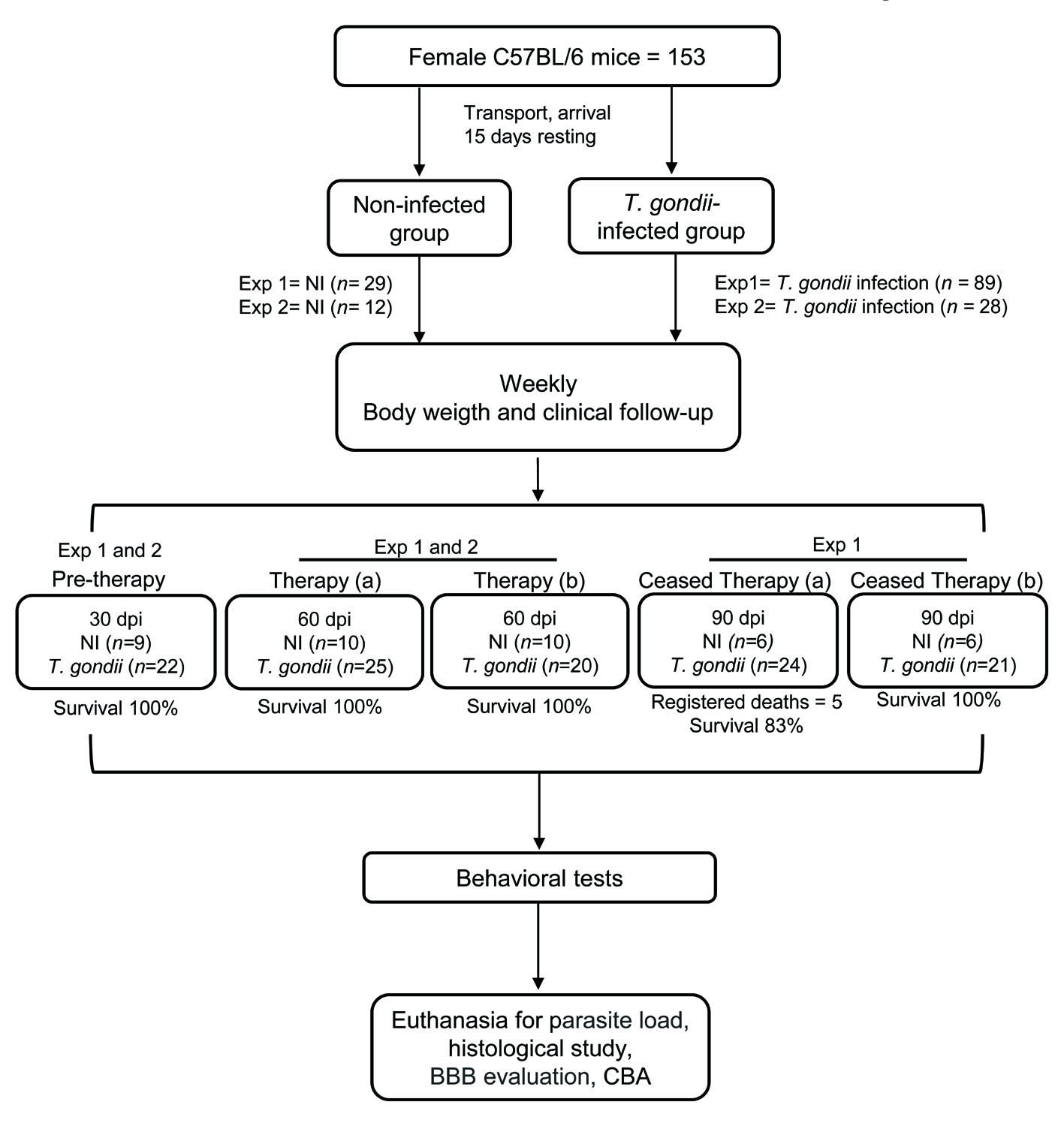

Supplement: Supplementary Figure 1 — Flow chart showing the experimental protocol with the experimental n in 2 (pre-therapy and therapy group) or 1 (ceased therapy group) independent experiments and survival. [file Image_1.tif]

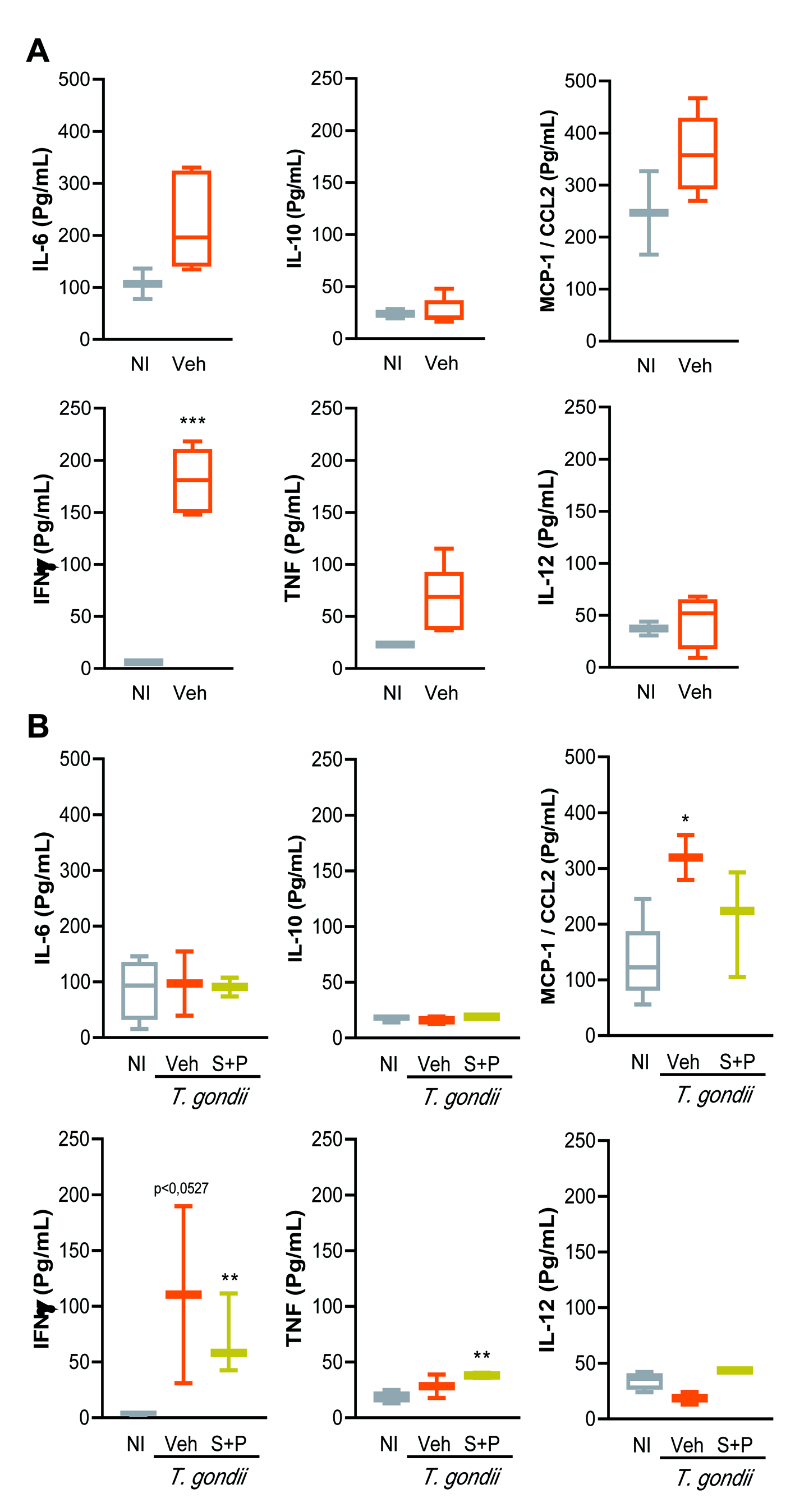

Supplement: Supplementary Figure 2 — Cytokine serum levels pre-Therapy and after Ceased Sulfadiazine plus pyrimethamine therapy. (A) Concnetrations of IL-6, IL-10, MCP-1/CCL2, IFNγ, TNF, IL-12 in serum of NI control mice and T. gondii-infected mice, at 30 dpi. (B) Concnetrations of IL-6, IL-10, MCP-1/CCL2, IFNγ, TNF, IL-12 in serum of NI control mice and Vehicle-treated and S+P-treated T. gondii-infected mice, at 90 dpi. Each experimental group consisted of 2-3 NI mice and 2-8 mice infected with T. gondii. Cytokine levels are shown in box and whisker charts, with medians, and minimun and maximun values shown by vertical lines. Data were analyzed using the Kruskal–Wallis H test followed by post hoc Dunn’s multiple comparisons tests and the ordinary one-way ANOVA followed by the Tukey post hoc test, with multiple comparisons (A, B) *p<0.05; **p<0.01; ***p<0.001comparing mice infected with T. gondii and NI control mice. [file Image_2.tif]
