# Supplementary material for: Sulfadiazine Plus Pyrimethamine Therapy Reversed Multiple Behavioral and Neurocognitive Changes in Long-Term Chronic Toxoplasmosis by Reducing Brain Cyst Load and Inflammation-Related Alterations
Source: Front Immunol. 2022 Apr 27;13:822567. doi: 10.3389/fimmu.2022.822567 (PMC9091718; doi:10.3389/fimmu.2022.822567)
Supplement: Supplementary file 3 [file Table_1.docx]

**Supplementary table S1**. Values obtained using the Shapiro-Wilk test.

|  |  | **Pre-therapy** |  | **Therapy** | |  | **Ceased Therapy** | |
| --- | --- | --- | --- | --- | --- | --- | --- | --- |
| Parameter | NI Control | Veh |  | Veh | SP |  | Veh | SP |
| Weight loss | 0 | 2 |  | 1 | 0 |  | 1 | 0 |
| *Neuromuscular function* |  |  |  |  |  |  |  |  |
| Muscle strength | 0.7351 | 0.2486 | 0.6895 | 0.926 | 0.9741 | 0.0359 | 0.1703 | 0.5165 |
| *Motor function* |  |  |  |  |  |  |  |  |
| Rearing | 0.1807 | 0.6017 | 0.0275 | 0.1333 | 0.2711 | 0.6745 | 0.8341 | 0.4165 |
| Immobility time (OFT) | 0.1184 | 0.882 | 0.356 | 0.4691 | 0.4178 | 0.6113 | 0.3661 | 0.916 |
| *Behavioral changes* |  |  |  |  |  |  |  |  |
| Anxiety | 0.6572 | 0.1376 | 0.1705 | 0.1901 | 0.7769 | 0.0352 | 0.2933 | 0.3823 |
| Depression | 0.4451 | 0.9532 | 0.5587 | 0.0841 | 0.6492 | 0.996 | 0.0884 | 0.1107 |
| Hyperativity | 0.3325 | 0.4679 | 0.3712 | 0.2525 | 0.5894 | 0.5726 | 0.0242 | 0.1647 |
| *Memory impairments* |  |  |  |  |  |  |  |  |
| Habituation memory | 0.5484 | 0.6729 | 0.2611 | 0.0975 | 0.4102 | 0.0879 | 0.8987 | 0.5068 |
| Aversive memory retention | NA | <0.0001 | NA | <0.0001 | <0.0001 | NA | 0.0009 | <0.0001 |
| Aversive memory consolidation | 0.0185 | 0.0059 | 0.0012 | <0.0001 | 0.0034 | <0.0001 | 0.7002 | 0.0027 |
| *Brain cyst* |  |  |  |  |  |  |  |  |
| Number | NA | 0.9842 | NA | 0.4002 | 0.0141 | NA | 0.0065 | 0.9414 |
| Neuropathological alterations |  |  |  |  |  |  |  |  |
| BBB integrity | 0.0388 | 0.0018 | 0.047 | <0.0001 | 0.0001 | 0.0135 | <0.0001 | 0.1237 |
| Cytokine regulation |  |  |  |  |  |  |  |  |
| IL-6 |  | 0.1628 | 0.6298 | 0.0041 | 0.0626 | 0.898 |  |  |
| IL-10 |  | 0.0562 | 0.1912 | 0.0043 | 0.0912 | 0.328 |  |  |
| MCP-1 |  | 0.9758 | 0.5016 | 0.3743 | 0.6992 | 0.372 |  | 0.7116 |
| IFNɣ |  | 0.4491 | 0.8429 | 0.4443 | 0.2622 | 0.4984 |  | 0.4169 |
| TNF |  | 0.3013 | 0.2869 | 0.0043 | 0.9803 | 0.694 |  |  |
| IL-12 |  | 0.4686 | 0.666 | 0.0692 | 0.0005 | 0.5848 |  |  |

The values marked in bold indicate non-normal data.

NA, not analyzed.
